# From Cell‐Derived Vesicles to Hybrid Nanovectors: Biological Membranes as Functional Blueprints for Gene Delivery

**DOI:** 10.1002/adhm.71640

**Published:** 2026-08-25

**Authors:** Clara Baldari, Claudia Leone, Gabriella Leccese, Claudia De Stradis, Alessandra Invidia, Concetta Quintarelli, Biagio De Angelis, Franco Locatelli, Giuseppe Gigli, Gabriele Maiorano, Ilaria Elena Palamà

**Affiliations:** ^1^ Department of Experimental Medicine University of Salento Lecce Italy; ^2^ Institute of Nanotechnology (NANOTEC) ‐ National Research Council (CNR) Lecce Italy; ^3^ Tecnomed Puglia ‐ Tecnopolo Per La Medicina Di Precisione (Biotech Lecce Hub) Lecce Italy; ^4^ Department of Mathematics and Physics “Ennio De Giorgi” University of Salento Lecce Italy; ^5^ Department of Clinical Medicine and Surgery Federico II University of Naples Naples Italy; ^6^ Department of Onco‐Haematology and Cell and Gene Therapy Bambino Gesù Children's Hospital IRCCS Rome Italy; ^7^ Department of Life Sciences and Public Health Catholic University of the Sacred Heart Rome Italy

**Keywords:** biomimetic nanovectors, gene delivery, hybrid vesicles, membrane‐derived vesicles, nonviral gene therapy

## Abstract

Nonviral gene therapy reached unprecedented clinical prominence, with the approval of lipid nanoparticles (LNPs). Yet, their success remains largely confined to a narrow range of applications and tissues, which reflects the inherent functional constraints based on formulation optimization to overcome intrinsically complex physiological barriers. In contrast, biological membranes evolved to integrate immune evasion, targeting, intracellular trafficking, and membrane fusion within a single functional interface. In this review, we provide a mechanistic comparison between membrane‐derived vesicles (MDVs) and hybrid biomimetic systems. We critically examine MDVs from different biological sources, highlighting their unique advantages for gene delivery applications. We also focus on hybrid systems, which merge the biological functionality of naturally derived membranes with the design control of synthetic nanovectors to address key limitations such as inefficient cytosolic release, poor targeting specificity, and transfection of hard‐to‐engineer cells. This review provides a biologically informed roadmap for designing efficient gene delivery vectors, proposing membrane‐integrated nanovesicles (NVs) as “living interfaces” for non‐viral gene therapy.

## Graphical abstract

Biologically derived and hybrid nonviral nanovectors are examined as distinct but convergent design approaches. Integrating synthetic components with biologically functionalized membranes allows efficient interactions with complex cellular environments. This perspective highlights how compositional and structural synergies drive functional specialization, offering new opportunities for overcoming delivery barriers in gene therapy.

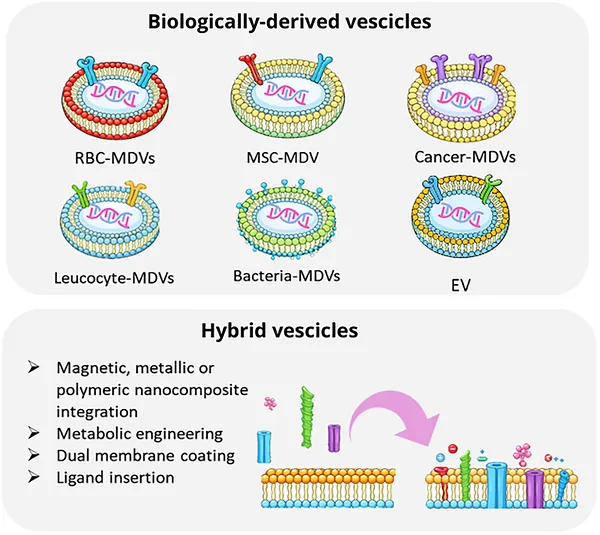

## 1 Introduction

Gene therapy is rapidly evolving from a conceptual therapeutic strategy into a clinically validated approach for treating a wide range of genetic, degenerative, and oncological disorders [1]. However, its success critically depends on the capacity of the genetic cargo to reach the target cells. Indeed, naked nucleic acids are vulnerable to serum nucleases, rapidly cleared by the kidneys, and subject to immunological activation [2]. To overcome these biological barriers, several delivery platforms have been developed, among which viral vectors have long represented the gold standard for efficient gene transfer. Although viral vectors are highly effective for delivering genetic material, their use is hindered by issues such as immunogenicity, potential genotoxic effects, complex manufacturing, and limited cargo capacity. These constraints inspired growing interest in nonviral delivery systems, especially lipid‐based nanoparticles (NPs), which in recent years have shown remarkable clinical impact, highlighted by the success of approved mRNA vaccines and other RNA‐based therapies [3, 4, 5]. Alongside the rapid progress of synthetic lipid nanoparticles, biomimetic delivery systems have emerged as a promising strategy to exploit the functional complexity of biological membranes for gene delivery, particularly through cell membrane camouflage, which endows nanoscale carriers with membrane‐derived biological functions. These nature‐inspired “camouflages” cover a wide variety of approaches, ranging from extracellular vesicles naturally released by cells [6], to NPs coated with cell membranes that carry over the characteristic molecular features of their source cells [7], and even to vesicles derived from bacterial membranes, which naturally provide their own immunomodulatory effects [8, 9, 10]. By retaining key membrane features such as lipid asymmetry, native proteins, glycocalyx patterns, and self‐recognition markers, these systems can modulate immune interactions, intercellular communication, and tissue‐specific targeting [11, 12, 13].

In parallel, increasing attention has been directed toward hybrid biomimetic delivery strategies and engineered biomimetic nanocarriers as platforms capable of combining biological complexity with the controllability of synthetic nanotechnology [14, 15, 16]. Despite numerous studies exploring individual membrane‐derived vesicles (MDVs), the field lacks a unified conceptual framework that integrates the different membrane sources comparing their gene delivery potential [15, 16]. The design of hybrid‐MDVs offers potential solutions to major bottlenecks of current nonviral vectors [15]. Here, we summarize recent advances in MDVs and hybrid‐MDVs for gene delivery, providing a comparative, mechanistic, and translationally oriented framework that reveals their collective potential as efficient platforms for clinical gene therapy.

## 2 Viral and Nonviral Gene Delivery Systems: Advances, Limitations, and Physiological Barriers

Despite early setbacks, significant progress in the treatment of human disease by gene therapy has been achieved and has offered patients an alternative to conventional therapies. The core of this progress is represented by the development of efficient and safe gene delivery vectors, which are broadly classified as viral or nonviral [17, 18]. Nowadays, an increasing number of viral vectors are under preclinical and clinical evaluation for the treatment of acquired and inherited diseases. Thanks to their innate ability to infect cells, they are efficient in intracellular nucleic acid delivery and can integrate therapeutic genes into the host genome, allowing long‐term expression [19]. One of the key advantages of the most used viral vectors in clinical trials, such as adenoviruses (Ad), lentiviruses, retroviruses, and adeno‐associated viruses (AAV), is the ability to escape endosomes, avoiding degradation within lysosomes [20].

However, their clinical application is limited by several intrinsic drawbacks, including strong immunogenicity, pre‐existing immunity in the population, risk of insertional mutagenesis, restricted cargo capacity, natural tropism toward specific tissues, and complex and costly manufacturing processes [19, 20, 21, 22, 23, 24, 25, 26].

The above‐mentioned challenges could be overcome by non‐viral gene delivery systems as safer alternatives. Nonviral vectors include both physical approaches, such as gene gun, electroporation, and magnetofection, and chemical nanocarriers [27]. Unlike viral vectors, they do not infect cells, but their effectiveness relies on physical‐chemical principles, making them platforms intrinsically safer and more versatile for gene delivery.

In particular, nanotechnology‐based delivery systems allow modularity, close control over size, charge, surface chemistry, and responsiveness to physiological signals, which can be engineered to optimize delivery efficiency, endosomal escape, and tissue specificity. Different nanovectors are under clinical evaluation, and some of them have already obtained approval. Among the most successful non‐viral platforms, LNPs have gained widespread attention, particularly after their clinical success in mRNA vaccines [28] (mRNA‐1273 and BNT162b2) [27, 29, 30], and are currently under extensive clinical investigation for cancer immunotherapy and infectious diseases [31, 32, 33].

Other emerging systems, such as polymeric and carbohydrate‐based, have also shown translational promise, particularly for RNA delivery and for crossing difficult biological interfaces such as the blood‐brain barrier (BBB) [34, 35]. Recent clinical advances, including LNP‐based RNA therapeutic strategies for pediatric brain tumors, further emphasize this potential and have already progressed to early regulatory approval for clinical testing (Food and Drug Administration (FDA) IND#BB‐19304) [36, 37].

Nonviral vectors remain limited by several intrinsic drawbacks that reduce their clinical performance despite their safety advantages over viral systems.

A first critical determinant of nanoparticle performance is their size‐dependent biodistribution. Particles smaller than ∼10 nm are rapidly eliminated through renal filtration [38], whereas those larger than ∼200–300 nm tend to accumulate in clearance organs such as the liver and spleen [39]. Even nanoparticles within the optimal size range remain exposed to additional biological barriers once in systemic circulation. Their overall delivery efficiency is limited by the need to avoid extracellular degradation and premature clearance, cross biological barriers, enter target cells, escape endosomal sequestration, and, in the case of DNA therapeutics, reach the nucleus [40]. Upon contact with blood components, NP surfaces rapidly adsorb plasma proteins, forming a dynamic protein corona driven by van der Waals, hydrophobic, and electrostatic interactions [41]. This acquired biological identity can alter NP’ stability and biodistribution, while promoting complement activation and immune recognition, ultimately shortening circulation half‐life and increasing nucleic acid degradation [42, 43]. To address these challenges, a range of surface engineering strategies has been developed to extend circulation time and enhance in vivo performance, including PEGylation, zwitterionic materials, and cell membrane‐based coatings [44]. Once they evade systemic clearance, NPs must extravasate from the bloodstream and penetrate the target tissue, encountering endothelial barriers that vary significantly across different organs. In most healthy tissues, tight endothelial junctions strongly limit NPs’ passage. In contrast, tumor tissues present a partially permissive environment due to their leaky vasculature and impaired lymphatic drainage, which can promote passive accumulation through the Permeability and Retention (EPR) effect [45, 46]. However, this effect is highly heterogeneous among tumor types and patients, and its efficacy is often reduced by elevated interstitial fluid pressure and dense extracellular matrix, both of which hinder deep tissue penetration [47]. As a result, active targeting strategies using antibodies, peptides, aptamers, or other ligands are increasingly used to improve specificity and uptake efficiency [38, 48]. In this context, one of the most challenging endothelial barriers for gene delivery is the BBB. Formed primarily by brain microvascular endothelial cells, connected by tight junctions, this complex barrier works in synergy with astrocytes’ end‐feet and pericytes to strictly regulate the passage of most macromolecules and foreign particles [49]. To bypass the BBB, NP functionalization strategies aim to exploit transport mechanisms, cellular interactions, and microenvironmental cues, including ligands or antibodies that bind to receptors abundantly expressed on the BBB [50], cell‐penetrating peptides (CPPs) [51], BBB‐crossing lipids [52], and biomimetic and cell‐derived coatings [53, 54].

Once inside the cell, NPs encounter additional intracellular challenges. Following endocytosis, materials traffic progressively from early to late endosomes and ultimately lysosomes, where acidic conditions and hydrolytic enzymes can degrade nucleic acids [55]. Endosomal escape remains one of the major bottlenecks in nonviral delivery, with only a small fraction (∼1%–4%) of internalized material successfully reaching the cytosol [15]. Stimuli‐responsive systems that exploit pH, redox, or enzymatic cues have therefore been developed to trigger controlled disruption of endosomal membranes and improve cytosolic release [56, 57]. For DNA delivery, nuclear import via nuclear pore complexes (NPCs, 9–10 nm diameter) represents an additional barrier, particularly in nondividing cells where nuclear envelope breakdown during mitosis cannot be exploited. In these cells, DNA entry into the nucleus relies mainly on NPC‐mediated transport and, for larger cargoes, on energy‐dependent mechanisms involving karyophilic proteins and nuclear localization sequences (NLS). Accordingly, several strategies have been developed to improve nuclear delivery, including SV40 enhancer sequences, NLS‐tagged DNA or vectors, and other nuclear‐targeting ligands [58, 59, 60]. Beyond the physiological barriers encountered in vivo, nonviral vectors are inherently limited by physicochemical constraints associated with their material composition and structural design. Some materials such as cationic lipids and polymers can improve condensation, cell uptake, and endosomal escape, but excessive positive charge may damage cellular membranes, causing significant cytotoxicity, especially at higher doses or when nondegradable materials are used [61, 62, 63]. A further problem is the difficulty in controlling intracellular trafficking and cargo release. If nucleic acids are bound too tightly, unpacking becomes inefficient; if binding is too weak, the payload may be lost before reaching the site of action [64, 65]. In addition, many non‐viral vectors show lower transfection efficiency compared to viral vectors [66] and limited tissue specificity, which can result in the therapeutic genes being expressed in non‐target organs, leading to toxicity [67].

In conclusion, a cascade of extracellular and intracellular barriers must be overcome to ensure a successful delivery of nucleic acids using NPs. By integrating biomimetic interfaces, active targeting, and stimuli‐responsive macromolecules, NPs can improve their stability in systemic circulation, cellular uptake, and cytoplasmic or nuclear release, ultimately maximizing therapeutic efficacy while minimizing off‐target effects.

## 3 Biomimetic Nanovector Design: Exploiting the Plasma Membrane to Improve Nonviral Gene Delivery

The multiplicity of extracellular and intracellular barriers described above suggests that improving non‐viral gene delivery requires more than incremental optimization of individual formulation parameters. In this context, biological membranes provide a particularly attractive source of inspiration, because they naturally integrate circulation stability, tissue penetration, cellular recognition, and intracellular trafficking within a single interface [68, 69]. These characteristics have inspired a continuum of biomimetic delivery systems, from simplified membrane‐inspired NPs to membrane‐derived vesicles (MDVs) that retain native cellular components [5, 7, 14]. Liposomes were originally conceived as simplified structural mimics of cellular membranes, consisting primarily of phospholipid bilayers. By contrast, LNPs, rather than reproducing membrane architecture, are highly engineered nanocarriers that recapitulate selected membrane‐associated functions, including colloidal stability, membrane interaction, cargo protection, and endosomal escape. Unlike conventional bilayer liposomes, ionizable LNPs are characterized by dynamic and heterogeneous internal organization, often containing amorphous or inverted hexagonal lipid phases generated by ionizable lipids and helper lipids, specifically engineered to promote pH‐responsive membrane destabilization and efficient endosomal escape [70, 71, 72]. MDVs directly retain key compositional features of their biological source, including lipid asymmetry, phospholipids, glycolipids, cholesterol, and a rich repertoire of proteins whose abundance and organization depend on cell type [69, 73]. This membrane organization is functionally important because it governs fluidity, curvature, rigidity, intracellular homeostasis and cell‐specific communication. Phosphatidylcholine (PC), present mainly in the outer layer, contributes to membrane fluidity and stability, whereas phosphatidylethanolamine (PE), phosphatidylserine (PS), and phosphatidylinositol (PI), enriched in the inner leaflet, support membrane curvature, remodeling, and fusion processes involved in intracellular trafficking. Several of these lipid‐mediated properties have inspired the design of helper lipids and fusogenic formulations used in LNP engineering. However, whereas biological membranes achieve these properties through tightly regulated lipid asymmetry, LNPs rely on tailored lipid compositions and dynamic internal phases to reproduce comparable delivery functions. Although LNPs can reproduce some membrane‐associated biophysical functions, they do not preserve higher‐order membrane features such as lipid rafts or lateral lipid segregation [15, 74, 75]. Another important functional analogy between synthetic and biological systems concerns surface shielding mechanisms. PEGylation is largely used in LNPs to reduce protein adsorption and to prolong circulation time, but it could induce anti‐PEG immune responses in case of repeated administrations. On the contrary, glycoproteins constituting the glycocalyx on the outer leaflet of the plasma membrane create a hydrated steric barrier conferring immune camouflage without triggering adverse reactions [76]. The negative charge of the plasma membrane surface, which derives from sialic acid residues in glycoproteins and phosphate‐containing headgroups [77, 78, 79], further enhances colloidal stability and modulates biodistribution. This model of endogenous charge influences organ tropism in an analogous manner to the selective organ targeting (SORT) strategy developed for LNPs [80], but through mechanisms physiologically codified rather than through synthetic design. Crucially, MDVs incorporate transmembrane proteins that introduce biologically active functions absent in synthetic LNPs. These proteins include receptors, transporters, adhesion molecules, and antigens that allow selective cell recognition and receptor‐mediated endocytosis [69, 73] without the necessity of functionalization with specific ligands. Some proteins associated with the plasma membrane facilitate endosomal evasion; for instance, annexins promote membrane destabilization and cytosolic cargo release [81]. This complexity is crucial for targeting cells such as primary T cells, in which rigid cytoskeletal and low basal endocytic activity could limit delivery vectors’ performance. By exploiting physiological membrane interactions and alternative trafficking pathways, MDVs could represent a more compatible strategy for efficient gene transfer in hard‐to‐transfect cells. As summarized in Table 1, synthetic LNPs reproduce selected physicochemical functions naturally performed by membrane lipids, whereas MDVs retain the molecular features from which these functions originate. Understanding this relationship between engineered and biological lipid systems provides a conceptual framework for the rational design of next‐generation gene delivery platforms.

**TABLE 1:** Comparison of major lipid classes used in LNP formulations, their role in nucleic acid delivery, and their biological membrane counterparts. Abbreviations: PEG, polyethylene glycol; PE, phosphatidylethanolamine; PS, phosphatidylserine; PC, phosphatidylcholine; PI, phosphatidylinositol; RBCs, red blood cells; MSCs, mesenchymal stem cells; EVs, extracellular vesicles; SORT, selective organ targeting; SIRPα, signal regulatory protein alpha.

Component/molecule	Chemical structure/type	Main function in LNPs	Natural analog in biological membranes and analogous function	Ref.
Ionizable cationic lipids	Amphiphilic molecules with ionizable amine groups (e.g., DLin‐MC3‐DMA, SM‐102, ALC‐0315)	Facilitate nucleic acid complexation and endosomal escape through pH‐dependent charge reversal	PE, PS. Both promote membrane curvature and fusion	[15, 74, 75]
Permanently charged cationic lipids	Quaternary ammonium headgroup, long hydrophobic tails (e.g., DOTAP, DODMA)	Form stable electrostatic complexes with anionic nucleic acids, promote endosomal escape	Positively charged peptides, aminolipids in microbial membranes. Both interact electrostatically with anionic biomolecules or membranes	[74, 75]
Fusogenic helper phospholipids	Zwitterionic phospholipids with saturated or unsaturated acyl chains and small headgroups (e.g., DSPC, DOPE)	Support fusogenic transitions and formation of nonlamellar phases; aid endosomal escape	PE. Maintain bilayer flexibility; promote fusogenicity	[68, 82]
Structural helper phospholipids	Zwitterionic phospholipids with saturated or mixed acyl chains (e.g., DOPC, POPC)	Stabilize the bilayers; tune fluidity and mechanical robustness	PC, PI. Maintain bilayer structure and lateral organization; a major component of the biological outer leaflet	[68, 83]
Anionic lipids	Negatively charged phospholipids (e.g., DOPS, DOPA)	Adjust surface charge, modulate protein adsorption	PS, PI. Mediate signalling and recognition	[68, 77, 78]
Cholesterol	Sterol with a rigid planar ring	Increases packing density, mechanical strength, and serum stability	Cholesterol (membrane component). Regulates fluidity, permeability and lipid raft formation	[15, 74, 75, 84]
β‐sitosterol/ phytosterols	Sterols with an additional ethyl group at C‐24	Modulate bilayer packing and shape; enhance fusion and transfection efficiency	Plant sterols, bacterial hopanoids. Tune membrane rigidity and permeability	[75, 84]
Sphingomyelin	Sphingosine backbone with amide‐linked fatty acid	Contributes rigidity and ordered packing	Sphingomyelin (outer leaflet lipid). Increases order and mechanical stability, participates in raft formation	[68, 76, 79]
Glycolipids	Carbohydrate headgroup plus lipid tail	Enhance targeting and reduce non‐specific immunogenicity by providing recognition motifs	Gangliosides, glycosphingolipids. Provide recognition motifs and stabilize glycocalyx‐like interfaces	[64, 67, 70, 73, 76, 79]
PEG‐lipid conjugates	Hydrophilic polymer chain covalently linked to lipid tail (e.g., DMG‐PEG2000, DSPE‐PEG2000)	Provides stealth and colloidal stability by forming a hydrated steric barrier	Glycoproteins/glycocalyx polysaccharides (e.g., sialic acid, glycosaminoglycans). Generate a steric and hydrated barrier, preventing opsonization and immune clearance	[76, 77, 78, 79]

## 4 Classification and Structural Design of Membrane‐Derived Vesicles

In this review, three main types of biomimetic NPs are described, each characterized by specific structural features optimized for gene cargo protection and delivery (Figure 1).

**FIGURE 1:**
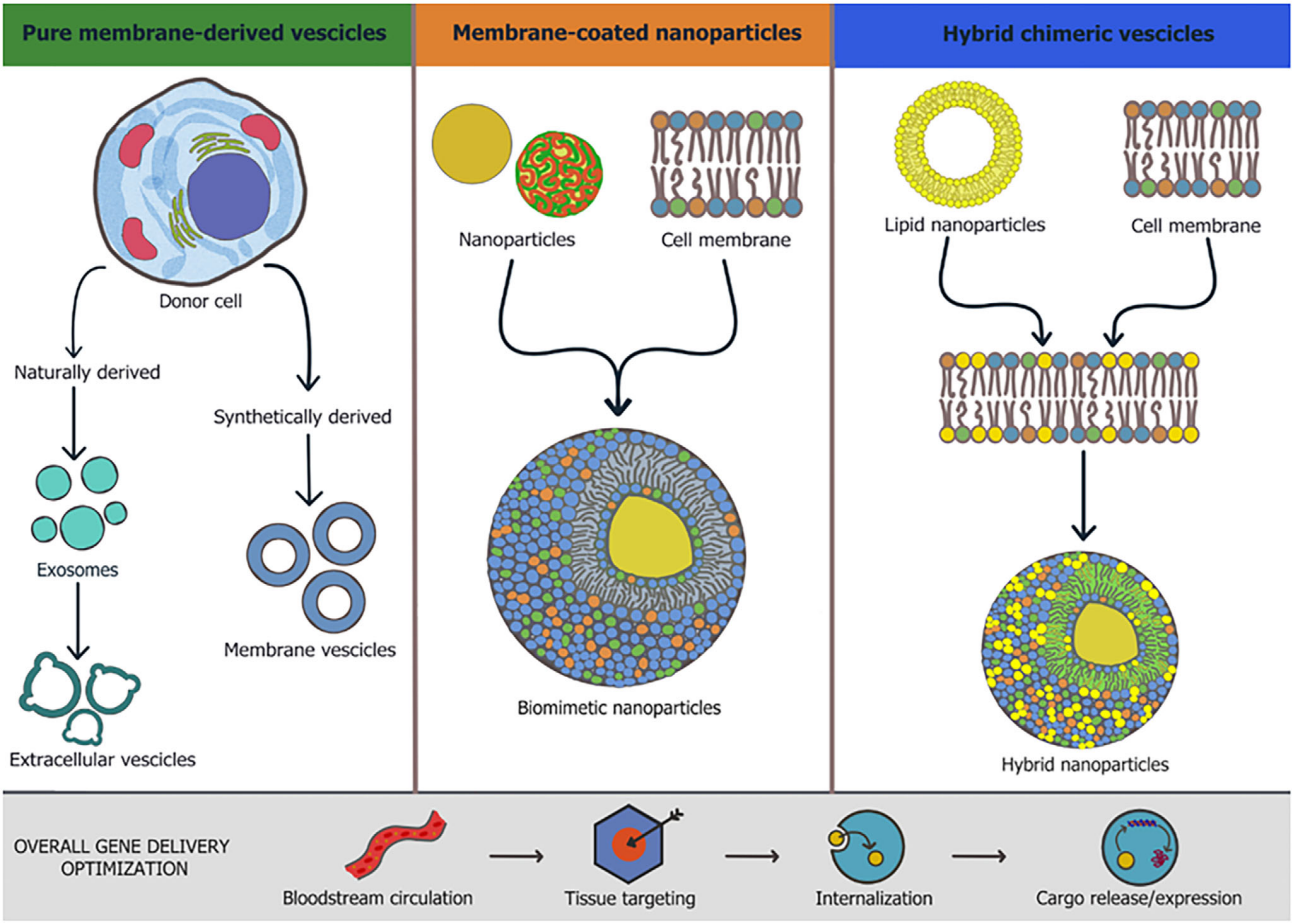
Classification of membrane‐derived gene delivery systems. Membrane‐derived vectors can be organized along a continuum of biomimetic complexity, ranging from pure membrane‐derived vesicles (PMVs) that preserve native membrane architecture, to membrane‐coated NPs (core‐shell systems), and hybrid chimeric vesicles that integrate biological membranes with synthetic lipid nanocarriers.

### 4.1 Pure Membrane‐Derived Vesicles

One of the biomimicry approaches involves the use of vesicles composed entirely of biological membranes. These include naturally occurring extracellular vesicles (EVs), such as exosomes and microvesicles [85], as well as cell membrane‐derived nanovesicles (CMDVs) synthetically generated from donor cells [86].

### 4.2 Membrane‐Coated NPs

Core‐shell biomimetic structures, instead, are typically made of a synthetic core and a cell‐membrane coating. The core, composed of organic or inorganic materials, is specifically engineered to condense nucleic acid payloads and to control cargo release [87]. This is subsequently coated with a cell‐derived membrane, creating a system in which cargo delivery functions are separated from biological interface properties [88]. By preserving native protein topology during the coating process, typically achieved via mechanical extrusion or sonication [89], and more recently also explored through emerging microfluidic approaches such as microfluidic squeezing and nanochannel‐based mechanoporation [90, 91], the NP moves beyond simple structural mimicry to actively acquire the cell's functional identity.

### 4.3 Hybrid Chimeric Vesicles

Unlike core‐shell systems, hybrid chimeric vesicles are built as a single, fully integrated membrane in which synthetic and natural components coexist within a continuous fluid‐mosaic architecture [92]. Rather than being spatially segregated, as in membrane‐coated NPs, synthetic and natural constituents are intermixed at the molecular level, enabling the simultaneous exploitation of the structural tunability, manufacturability, and cargo‐loading capacity of LNPs together with the complex biological functions of native cell membranes [93].

## 5 Cell‐Mimicking Nanoparticles: Bridging Biological Function and Therapeutic Gene Delivery

### 5.1 EVs as Endogenous Nanovector for Gene Delivery

Following the structural classification provided above, EVs represent the most sophisticated example of pure biological interfaces [94]. EVs are nanoscale, lipid membrane vesicles that contain a broad range of biologically active molecules such as DNA, mRNA, microRNA, metabolites, proteins, lipids, and sugars [86]. Actively secreted by most cell types (Figure 2), EVs serve as key players in intercellular communications through the membrane fusion process from parental to other cells [95].

**FIGURE 2:**
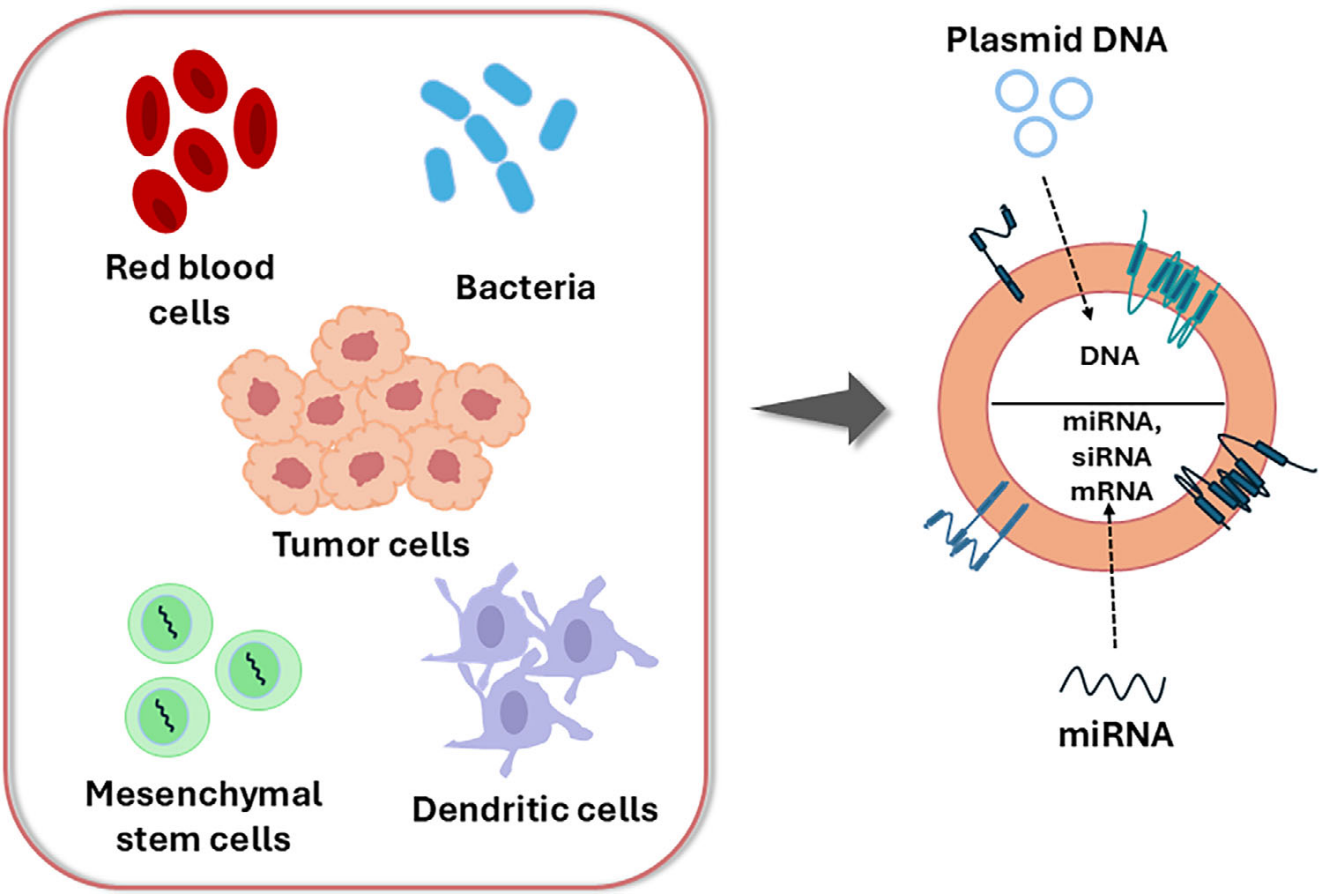
Schematic representation of EVs derived from different source cells (red blood cells, bacteria, tumor cells, mesenchymal stem cells, and dendritic cells) used as biological carriers or sources for delivering therapeutic nucleic acids, including plasmid DNA, mRNA, and miRNA.

Because of some properties like suitable size, high biocompatibility, high stability, low immunogenicity, low cytotoxicity, long half‐life in circulation, the ability to safeguard cargo against deterioration, and the capacity to cross the BBB and placental barriers, EVs are now regarded as natural nanostructured gene delivery systems for biomedical applications [96].

Although EVs are naturally released into the extracellular environment, their production can be enhanced through optimized culture conditions, bioreactor systems, physical stimulation, or pharmacological modulation of cellular pathways involved in vesicle biogenesis [97, 98, 99, 100, 101]. These approaches are particularly attractive for gene delivery applications, as EVs retain many structural and functional characteristics of the parental cell membrane, including membrane proteins and lipids involved in cellular recognition, targeting, and intracellular trafficking. Furthermore, EVs possess intrinsic mechanisms which facilitate endosomal escape, potentially improving intracellular gene delivery.

Despite these advantages, challenges remain regarding large‐scale production, loading efficiency, targeting specificity, and the incomplete understanding of intracellular trafficking pathways following uptake by recipient cells [94]. Consequently, the choice of parental cell source has become a critical factor in EV engineering, as it directly influences biodistribution, targeting capability, and therapeutic performance. Dendritic cell‐derived extracellular vesicles (DC–EVs) stand out among EV‐based gene delivery systems because they retain membrane proteins that are directly involved in antigen presentation and immune cell signaling, such as MHC molecules, CD80/CD86, and Intercellular Adhesion Molecule‐1 (ICAM‐1). This gives them a natural ability to interact selectively with immune cells and to exert intrinsic immunomodulatory effects, which can be leveraged to couple nucleic acid delivery with either immune activation or tolerance. In addition, DC–EVs show high biocompatibility, low toxicity, and relatively low immunogenicity. They can also be engineered to load therapeutic RNA or DNA and to display targeting ligands on their surface [102, 103, 104].

Tumor‐derived extracellular vesicles (TD–EVs) have attracted attention as gene delivery carriers because they reflect many of the features of the tumor cells they come from. In particular, their surface composition appears to favour recognition and uptake by cells of the same tumor type. This homotypic interaction can be useful for improving targeting and reducing non‐specific distribution. At the same time, these vesicles are naturally involved in communication within the tumor microenvironment (TME), which supports the transport of nucleic acids and helps shield them from degradation in extracellular conditions [105, 106]. However, their endogenous oncogenic cargo raises safety concerns that may limit their direct therapeutic application [107].

According to the literature, mesenchymal stem cells (MSCs) have been reported to release more EVs than many other cell types, such as myoblasts or human embryonic kidney cells [108]. MSCs‐derived exosomes can replicate the paracrine effect and biological activity of MSCs [109], making them able to efficiently deliver genetic cargo [110]. To address the tissue‐specific delivery challenge, EXOs derived from conjunctival epithelial cells (CECs) were used for encapsulation of siRNA targeting the NFKBIZ gene [111]. The obtained vesicles maintain molecular characteristics on their surface, as well as the targeting capability of parent cells, enabling efficient and selective homing to corneal tissue. As a result, the significant silencing of the NFKBIZ gene leads to reduced secretion of pro‐inflammatory cytokines, restores the inflammatory balance on the ocular surface, and ultimately achieves a robust therapeutic effect in a dry eye disease (DED) mouse model [111].

Red blood cell (RBC) derived EVs offer a set of advantages. Naturally generated during ageing to remove harmful components like denatured haemoglobin, band 3 neoantigens, and immunoglobulin G [111], EVs are enriched in glycophorin A and lipid raft proteins but depleted in actin compared to RBCs. Their ability to preserve microRNAs (miRNAs) [111, 112] and the function of source cells position RBC‐EVs as promising for gene delivery, being safely isolated from blood banks or patients’ own blood, devoid of DNA, and capable of preserving RNA stability and function in recipient cells, including cancer contexts [113].

Collectively, these observations position EVs as biologically evolved NVs that integrate membrane complexity, intrinsic targeting, and intracellular trafficking. While challenges due to scalability, targeting selectivity, and nucleic acid loading efficiency remain, EVs could be an efficient tool for gene delivery.

### 5.2 Cell Membrane‐Derived Vesicles

Another recent approach that has been developed consists of the fabrication of MDVs obtained directly from the native cell membranes and used as a mimetic nanocoating for a gene delivery system [114]. By exploiting this biomimetic approach, the adverse effects related to the “nonself” property of synthetic carriers may be overcome as membranes derived from autologous cells present endogenous self‐markers and are therefore recognised as self by the immune system [115]. Thus, by straightforward membrane coating, various biological functions and targeted ability without further modification can be achieved.

#### 5.2.1 Blood Cell Membrane‐Derived Vesicles

Blood cell‐MDVs represent one of the most extensively investigated classes of biomimetic vectors for gene delivery, owing to their intrinsic biocompatibility, biodegradability, and low immunogenicity. By retaining source membrane features, these systems preserve the biological identity of their parent cells.

##### 5.2.1.1 RBC–MDVs

Among them, RBC‐MDVs are one of the most widely used bioactive carriers. Primarily because of the retained cell‐membrane proteins on the RBC surface, such as CD47, which promotes a long circulation time and cargo protection from the immune system by acting as a “don't eat me” signal [116]. RBC camouflage has been successfully exploited to improve gene delivery efficiency. For example, Hao et al. developed RBC‐MDVs encapsulating a pZNF850 plasmid, demonstrating high cell viability of treated endothelial cells (ECs) and a high transfection efficiency [115]. The phagocytic rate dramatically decreased, indicating high immune‐escaping ability due to the RBC membrane coating, which reduces the susceptibility to macrophage uptake. In addition, the high content of RBC membrane showed the most systematic circulation in vivo related to the cell membrane camouflaged gene delivery system, which is of great significance for the in vivo application [115].

##### 5.2.1.2 Leukocytes‐MDVs

Leukocytes, another actor of the blood system, are also useful to generate leukocyte‐MDVs that can delay uptake by the mononuclear phagocyte system, preferentially bind inflamed endothelium, and facilitate transport across the endothelium, avoiding the lysosomal pathway [117]. Krishnamurthy et al. fabricated leukocyte‐MDVs that retain surface proteins such as CD45, CD3z, lymphocyte function‐associated antigen 1 (LFA‐1), and macrophage‐1 antigen (Mac‐1) adhesive proteins, which play a role during vascular extravasation [118]. Moreover, this coating can activate the ICAM‐1 pathway, leading to VE‐Cadherin phosphorylation and the disruption of endothelial junctions between the cells to increase tumor vascular permeability [119]. In vivo studies showed that this approach improved the targeting properties, enhanced tumor perfusion, and promoted firm adhesion to the tumor vasculature, supporting the therapeutic efficacy of leukocyte‐MDVs [119]. These properties allow leukocyte‐MDVs to bypass lysosomal sequestration and improve intracellular cargo delivery.

##### 5.2.1.3 Monocyte‐MDVs

Monocyte‐MDVs further extend this targeting paradigm by exploiting adhesion molecule overexpression in the TME. In previous studies, breast cancer cell lines have been shown to overexpress vascular cell‐adhesion molecule 1 (VCAM‐1), which binds to α4β1 integrin of circulating monocytes [118]. Specifically, CD49d, a part of the heterodimeric integrin α4β1, binds to VCAM‐1, making monocyte‐MDVs able to improve cancer cell targeting and enhance intracellular uptake [120].

##### 5.2.1.4 T‐Cell‐MDVs

Among immune cell‐MDVs, T‐cell‐MDVs have attracted increasing attention for their dual role in gene delivery and immunostimulation. Unlike their living counterparts, T‐cell‐MDVs do not respond to the immunosuppressive molecules in the TME. Instead, by retaining key membrane proteins, they can help restore the cytotoxic functions in cytotoxic T lymphocytes (CTLs) by blocking immune checkpoint interactions (*e.g*., PD‐L1) and scavenging immunosuppressive molecules (*e.g*., TGF‐β1) via PD‐1 or TGF‐β1R proteins on T‐cell‐MDVs, respectively [94]. Notably, cell‐MDVs exhibit higher therapeutic efficacy than immune checkpoint blockades such as anti‐PD‐L1 antibody, likely because their antitumor activity is not restricted by tumor antigen specificity [121]. T‐cell‐MDVs can accumulate in tumors by exploiting two different mechanisms: passive extravasation via the EPR effect and active adhesion to cancer cells and inflamed endothelium within the TME [119]. This targeting ability is mainly mediated by adhesion molecules retained on the T‐cell membrane, including LFA‐1, Mac‐1, and P‐selectin glycoprotein ligand‐1 (PSGL‐1), which bind to ICAM‐1 and selectins overexpressed on inflamed tumor endothelium [122]. In particular, LFA‐1 promotes efficient tumor accumulation, reducing off‐target distribution and improving targeting efficiency [122].

In vivo T‐cell‐MDV injection enhanced CD8^+^ IFN‐γ^+^ T cell and CD8^+^ Granzyme B^+^ T cell levels in the tumor tissues, suggesting a strong antitumor immune activity. Their therapeutic efficacy was further enhanced when combined with cytotoxic T lymphocyte antigen‐4 (CTLA‐4) blockade, which promoted CTL proliferation and reduced T‐cell exhaustion in draining lymph nodes [121].

##### 5.2.1.5 Macrophages‐MDVs

Macrophages‐MDVs further contribute to the versatility of blood cell‐based biomimetic vectors. Their targeting ability is largely mediated by surface receptors such as integrins, Mac‐1, and colony‐stimulating factor 1 receptor (CSF1R) [123]. For example, Gong et al. developed biomimetic membrane camouflaged poly (lactic‐co‐glycolic acid) (PLGA) NPs starting from macrophages and cancer cell plasma membranes for siRNA (siFGL1) encapsulation. The resulting siFGL1 delivery can silence the related FGL1 gene, inducing T‐cell‐mediated immune responses and enhancing antitumor immunity [124]. Additionally, combining the PD‐L1/programmed death 1 signalling blockade and FGL1 gene silencing, a high synergistic therapeutic efficacy was obtained against breast cancer, both in vitro and in vivo [125].

##### 5.2.1.6 Platelet‐MDVs

Platelet‐MDVs (PLT‐MDVs) represent another powerful platform gaining attention during the past decades because of PLTs’ capabilities, like immune evasion, sub‐endothelium adhesion, and pathogen interactions [126, 127, 128]. Among surface proteins, the PLT membrane also expresses unique surface ligands for active targeting on tumor cells, damaged vasculature, and pathogenic bacteria [129]. These interactions are mediated by tumor‐associated molecules such as PSGL‐1, podoplanin, ADAM‐9, and fibrinogen/αvβ3, which interact with PLT receptors including CLEC‐2, P‐selectin, integrin α6β1, and integrin αIIbβ3 [130]. As a result, PLT‐MDVs can exploit the natural PLT‐tumor crosstalk to achieve enhanced accumulation within cancer tissues.

PLT membrane domains, enriched in cholesterol, saturated lipids, and sphingolipids, serve as functional platforms that regulate various cellular processes, including signal transduction, cell adhesion, and membrane trafficking [131].

PTL‐MDVs show colloidal stability due to the stabilizing effect of PLT's hydrophilic surface glycans and reduced macrophage uptake due to the expression of CD47 on PLT's surface. Additionally, these kinds of MDVs prevent complement activation in autologous human plasma. In fact, 90% of particles showed that 30 min after intravenous injection in vivo, they were distributed in tissues instead of being degraded by the immune system [132].

These properties have been exploited in a PLT membrane‐coated porous metal‐organic framework (MOF) for siRNA delivery, achieving efficient tumor‐targeting and siRNA release into the cytosol upon dissociation within the endosomal compartment, and allowing gene silencing and tumor growth inhibition [133]. Considering eventual use in human patients, using PLT membrane sourced from universal donors would largely reduce limitations about the long‐term immunogenicity of the NVs [132, 133]. Collectively, these features position PLT‐MDVs as attractive carriers for nucleic acid delivery, integrating immune evasion, tumor‐targeting capability, and controlled intracellular cargo release.

Blood cell membrane biomimicry endows NPs with native‐like surface chemistry, conferring prolonged circulation, immune evasion, and precise targeting capabilities, as exemplified in Figure 3.

**FIGURE 3:**
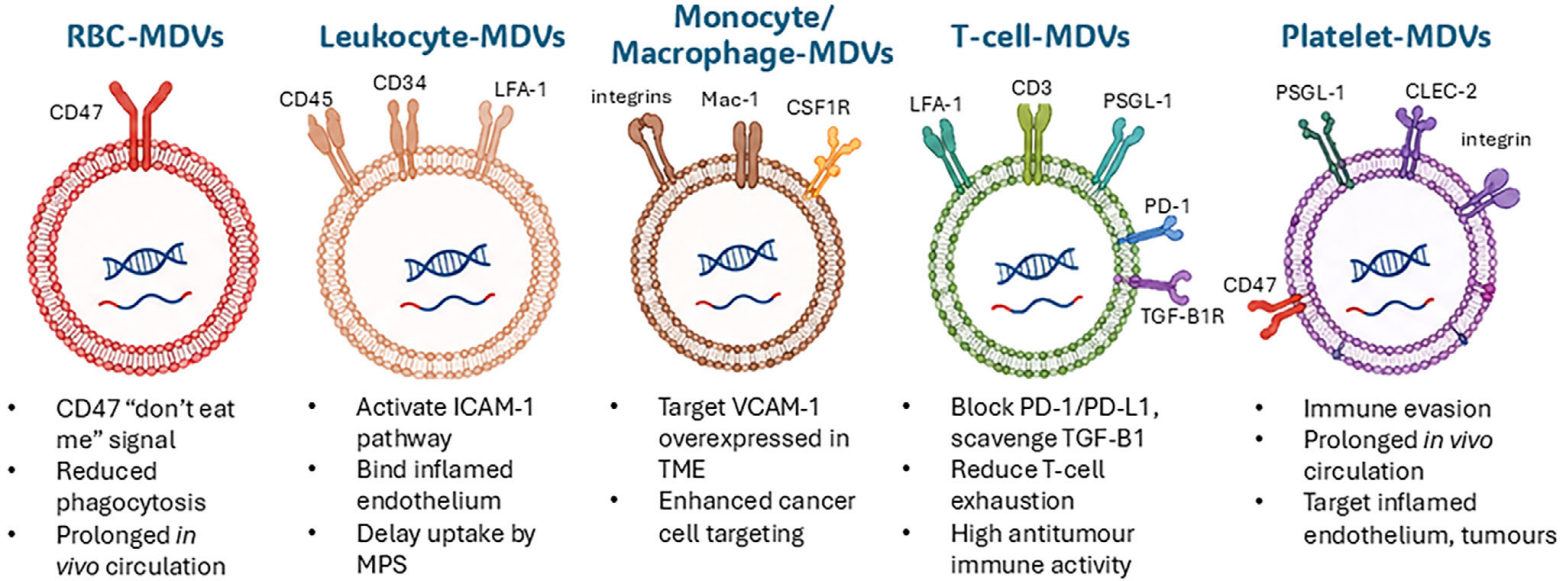
Blood cell membrane‐derived vesicles as nucleic acid delivery vectors for prolonged circulation and targeting and their parental cell‐acquired advantages.

#### 5.2.2 MSC‐Derived Vesicles Homing Ability

Among various bioinspired strategies, MSC–MDVs are attractive because of their homing capabilities through a multistep process toward chemokine gradients of the pathological microenvironment [134]. First, MSCs adhere to the vasculature by selectins/CD44 binding, then MSCs bind chemokines through C‐X‐C chemokine receptors [135, 136, 137]. During the arrest phase, integrins such as very late antigen‐4 (VLA‐4) facilitate adhesion to endothelial P‐selectin and VCAM1 [108, 138]. This is followed by diapedesis/transmigration, where MSCs extravasate and reach the damaged tissue [139, 140, 141]. Importantly, the expression of key homing receptors is culture‐dependent, highlighting the need for controlled expansion protocols to preserve targeting efficiency [137].

Leveraging this migration behaviour, MSC‐MDVs have demonstrated their potential to deliver therapeutic nucleic acids in multiple disease models. Examples include siRNA‐loaded MSC‐MDVs inducing cell death in mice with prostate cancer, and at the same time enabling magnetic resonance imaging (MRI) of tumor regions in vivo [142, 143]. Similarly, Bose et al. developed CXCR4‐containing bioengineered human adipose‐derived stem cell membrane‐functionalized NVs (BSMNCs) in order to provide strong targeting and to also boost their retention time in ischemic hindlimb tissue [144]. They functionalized PLGA NPs loaded with vascular endothelial growth factor to greatly promote the NVs’ penetration across the inflamed endothelial cell barrier. Furthermore, BSMNCs dramatically reduce the uptake by phagocytes in J774 (murine) and THP1 (human) macrophages compared to NVs without functionalization [144]. Other designs, such as hUC‐MSC membrane‐coated manganese dioxide (HMnO_2_) with TAT peptide modification, demonstrated the active targeting capacity of MSCs by tumoritropic accumulation in the malignant regions, hence preventing tumor development, recurrence, and metastasis [145]. Since MSCs frequently exhibit insufficient uptake at lesion sites, Wang et al. exacerbate the targeted homing properties of MSCs through a cutting‐edge tactic to increase the effectiveness of diabetic nephropathy (DN) therapy. They combined magnetic targeting to guide NP‐labelled placental MSCs to the kidney region in DN models. It was demonstrated that this targeting ability was crucial in lowering glomerular damage and albuminuria, which improved renal function more effectively than therapies using NP‐labelled MSCs without magnetic guidance or non‐guided MSCs [146]. Collectively, these examples illustrate how the natural migration, targeting, and immune evasion properties of MSC membranes can be leveraged to achieve precise and efficient gene delivery.

#### 5.2.3 Cancer Cell Membrane‐Derived Vesicles for Targeted and Immune‐Responsive Gene Delivery

Cancer cell plasma membranes expose a variety of adhesion molecules involved in tumor progression and metastasis dissemination, through both heterotypic (among cancer cells and other cell types) or homotypic (among similar cancer cells) adhesive contacts [142]. These molecules include integrins, selectins, E‐cadherins, Thomsen–Friedenreich (TF) antigens, and members of the immunoglobulin superfamily (Ig‐SF) [147]. Among them, TF‐antigen and E‐cadherin have been particularly associated with the homotypic aggregation of circulating tumoral cells [147].

By preserving these membrane‐associated recognition cues, cancer‐MDVs inherit biological functions that can be directly exploited for gene delivery.

In particular, cancer‐MDVs display a dual therapeutic role. First, they retain the homotypic recognition mechanisms of their parental tumor cells, promoting preferential accumulation within tumor tissues. Second, they retain tumor‐associated antigens (TAAs), which allow them to interact with antigen‐presenting cells and contribute to the activation of anti‐tumor immune responses. Moreover, the frequent overexpression of CD47 supports immune evasion and prolongs circulation time, further promoting tumor accumulation [148, 149, 150].

The immunostimulatory potential of cancer‐MDVs has been demonstrated in several studies. Fang et al. synthesized mannose‐modified cancer cell membrane NPs (CCM‐NPs) starting from the MDA‐MB‐435 melanoma cell line, which promote the interaction with DCs, stimulating their maturation and consequently enhancing anti‐tumor immune response [151].

Building on the same concept, Li et al. loaded siRNA encoding for Stat3 antigen into cancer‐MDVs. In this case, the membrane did not merely serve as a passive carrier but acted as an immunological component while delivering the therapeutic cargo. This dual role allows effective gene silencing, while also enhancing DC uptake and stimulating cytokine production, ultimately promoting immune cell recruitment within the TME [150].

Beyond immune activation, the intrinsic tumor tropism of cancer‐MDVs has also been exploited to improve therapeutic gene delivery [152]. Mesnil and Yamasaki used cancer‐MDVs to encapsulate the herpes simplex virus thymidine kinase plasmid (pHSVtk) as a therapeutic gene. The high transfection efficiency allowed the expression of HSVtk, which can phosphorylate ganciclovir, which can be transferred to surrounding tumor cells through gap junctions, causing apoptosis and enhancing the therapeutic effects [153].

Cancer‐MDVs transform characteristic features of tumor biology into functional advantages for gene delivery. By combining intrinsic tumor targeting with immunomodulatory activity, they represent one of the most biologically active membrane‐derived platforms currently available for cancer gene therapy. Despite their promising properties, the clinical applicability of cancer‐MDVs faces safety issues. Because the membrane source originates from malignant cells, residues of tumor components or immunogenic debris may risk off‐target inflammatory response [87].

## 6 Borrowing From Bacteria: Smart Membrane System for Gene Delivery

Among MDVs for gene delivery, bacterial membrane vesicles (BMVs) have emerged as versatile and immunologically active platforms for gene delivery. Outer membrane vesicles (OMVs) are EVs naturally secreted by bacteria. In Gram‐negative species, OMVs are typically 20–250 nm in diameter [154], whereas Gram‐positive bacteria, first reported to release EVs in 2009 from *Staphylococcus* [155], produce smaller vesicles (20–100 nm) that bud from the cytoplasmic membrane because they lack an outer membrane [156]. The composition of OMVs depends on bacterial species and physiological conditions. Gram‐negative OMVs contain lipoproteins, membrane proteins, cytoplasmic proteins, and other components involved in environmental adaptation [157]. While gram‐positive are enriched in cytosolic proteins, nucleic acids, and secreted factors, with cargo composition influenced by bacterial growth state [158]. The biological function of OMVs, ranging from gene transfer to cell communication, together with their inherent stability, ability to protect cargo from enzymatic degradation, and capacity for molecular transport, makes them a promising tool for gene and drug delivery [159, 160, 161]. The intrinsic immunogenicity of OMVs, mainly due to pathogen‐associated molecular patterns (PAMPs) such as lipopolysaccharide (LPS), peptidoglycan fragments, and bacterial lipoproteins, activates innate immune receptors (e.g., Toll‐like receptors—TLRs), facilitates cellular uptake and endosomal escape, and can synergize with nucleic acid therapeutics, particularly in cancer immunotherapy [162, 163, 164]. Moreover, OMVs can be modified to present immunostimulatory molecules, including TLR ligands, cytokines, or co‐stimulatory factors, immune checkpoint inhibitors, or anti‐angiogenic molecules, enabling simultaneous gene delivery and remodelling of the TME [165, 166]. Several studies have demonstrated the therapeutic potential of engineered OMVs. Cheng and colleagues [150] developed hyaluronidase (HAase) expressing, 3‐aminophenylboronic acid (PBA)‐functionalized *E. coli*‐derived OMVs loaded with CD73‐targeting siRNA (Figure 4A), which enhanced tumor penetration, reduced immunosuppressive adenosine production, promoted immune cell activation, and inhibited tumor growth (Figure 4B). Cargo loading strategies can rely on passive energy‐dependent diffusion, as in the above‐mentioned study, or on active methods such as electroporation, ultrasonication, and co‐extrusion, which transiently disrupt vesicle membranes to facilitate molecular entry [167]. One of the earliest demonstrations of genetically engineered OMVs for targeted gene delivery in cancer therapy was reported by Gujrati et al. [9]. They engineered HER2‐targeted OMVs carrying KSP siRNA, achieving selective gene silencing and tumor inhibition in HER2‐positive breast cancer models [9]. In a more recent study, OMVs from *E. coli* and *Lactobacillus reuteri* were electroporated with an enhanced green fluorescent protein (EGFP)‐coding plasmid DNA, synthetic EGFP RNA, and pre‐Cel‐miR‐39, and successfully delivered their cargo to microglial cells [168]. Additional strategies include cationic OMVs co‐delivering doxorubicin (DOX) and CD47‐targeting siRNA for synergistic chemo‐immunotherapy [169]. To overcome inefficient cargo loading and instability, a key limitation of current OMV systems, Wang et al. developed a modular, multifunctional OMV‐based CRISPR delivery platform. Using *E. coli*‐derived OMVs, the authors fused dCas9 with the outer membrane protein cytolisin A (ClyA), enabling self‐assembly and encapsulation of plasmid DNA directly within OMVs during bacterial growth. OMVs loaded with genes encoding CXCL9 and IL‐12 triggered T‐cell chemotaxis, activation, and finally resulted in the inhibition of tumor growth [170]. Beyond OMVs, bacterial protoplast‐derived nanovesicles (NVs) have recently emerged as an alternative platform [171] for gene delivery. Generated after enzymatic removal of the bacterial cell wall and outer membrane, these vesicles are largely free from endotoxins, while offering higher production yield, improved cargo loading efficiency, and enhanced biosafety compared with conventional OMVs [171, 172]. The growing number of studies on the use of BMVs for gene delivery underlines their potential, which includes biomimetic functionality, customizability, and scalable production. However, therapeutic application remains limited by challenges in production and isolation, variability in size and composition, restricted cargo capacity, and potential immunogenicity [166]. Ongoing efforts aim to address these limitations and improve BMV‐based cancer therapies.

**FIGURE 4:**
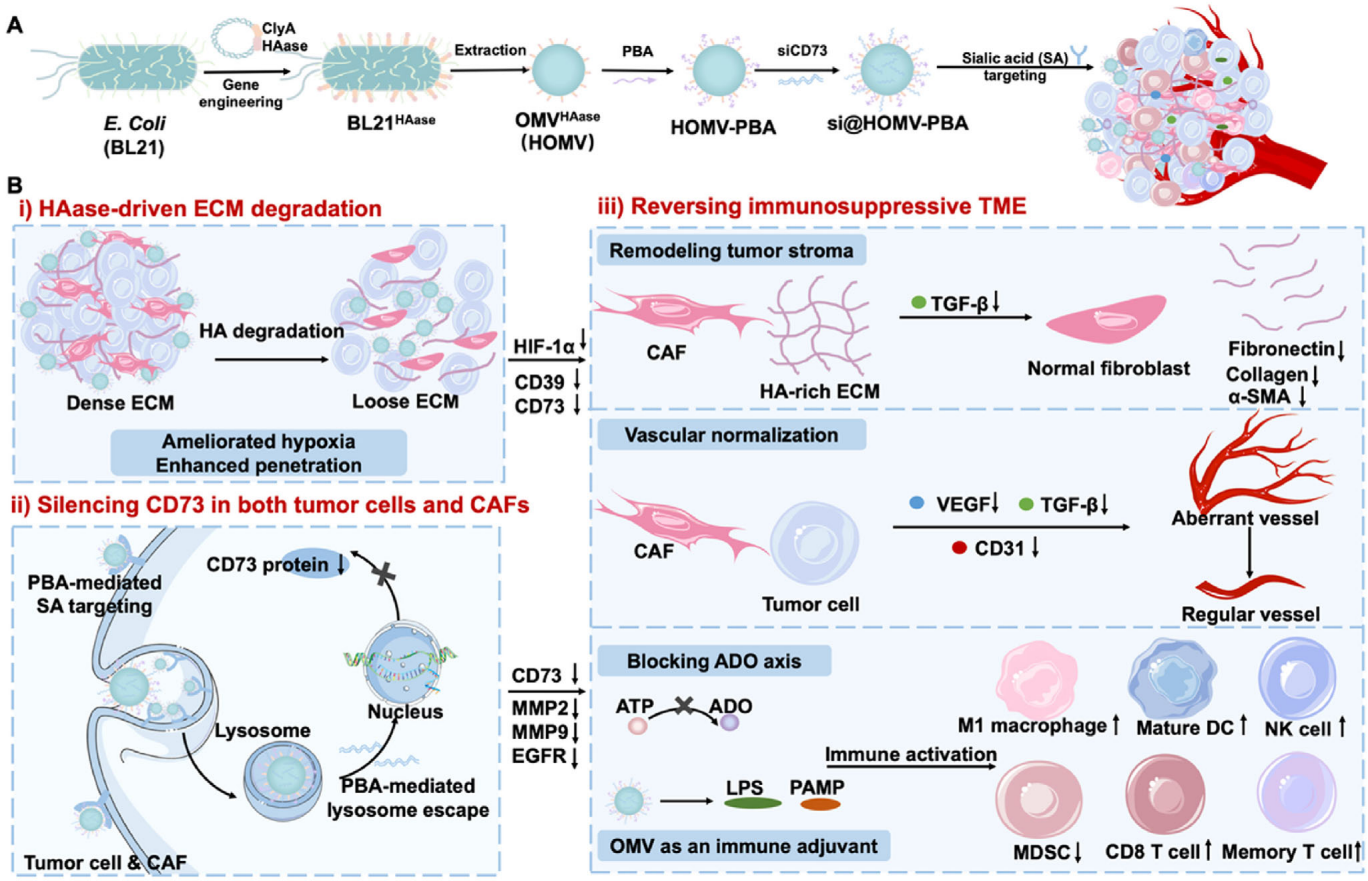
Schematic illustration of (A) si@HOMV‐PBA synthesis and (B) mechanism of action. Reproduced with permission [173]. Copyright 2026, Elsevier.

The main types of MDVs currently used for nucleic acid delivery are summarized in Table 2.

**TABLE 2:** Comprehensive overview of MDVs used for therapeutic nucleic acid delivery. The table summarizes seven distinct carrier types: Extracellular vesicles (EVs), red blood cell‐mimetic (RBC), leukocyte‐mimetic, platelet‐mimetic, cancer cell‐derived, mesenchymal stem cell‐derived (MSC), bacterial‐derived, and hybrid systems, their cargo capacity, biological targets, and key therapeutic advantages. Each system leverages specific biophysical properties to enhance biocompatibility, cellular targeting, and immune evasion, making them promising platforms for cancer therapy and treatment of inflammatory and ischemic diseases.

Type of MDVs	Cargo	Target	Advantages
EVs	DNA, mRNA, microRNA	Cancer therapy	High biocompatibility and stability Low immunogenicity Ability to cross the blood‐brain and placental barriers [85, 94, 96, 97, 98, 99, 100, 101, 174, 175]
RBC	miRNAs, pDNA	Liquid and solid cancers	Non‐immunogenicity Endosomal escape Safety [113, 115, 116]
Leukocyte	siRNAs	Tumor tissues	Enhancement of tumor vasculature permeability Prevention of the lysosomal pathway High accumulation in tumors [117, 118, 119]
Platelet	Synthetic siRNA	Cancer tissue, ischemic diseases	Prolongation of circulatory half‐life Enhancement of NPs’ pharmacokinetic profile Prevention of particle‐induced complement activation [126, 127, 128, 131, 132, 176]
Cancer cell	Therapeutic siRNA, HSVth plasmid	Cancer cells	Specific accumulation in TME Immune evasion Homotypic targeting [87, 142, 143, 147, 148, 151, 152]
MSCs	DNA, RNA, siRNAs	Tumors, inflammatory diseases	Homing capabilities in damaged tissues Immune evasion properties Retention in ischemic tissue [134, 135, 136, 140]
Bacterial	DNA, siRNA, CRISPR/Cas9	TME	PAMPs adjuvancy Endosomal escape Immune activation Scalable [154, 159, 160, 163, 164, 166]
Hybrid	Drugs, siRNA, CRISPR/Cas9	Cancer cells	Multiple targeting capabilities Enhancement of immunostimulatory properties Reduced off‐target effects [92, 166, 177]

## 7 Merging Biological Complexity With Synthetic Precision by Hybrid MDVs

Hybrid biomimetic NVs can be considered the most advanced convergence between the versatility of synthetic platforms and the functionality and complexity of natural MDVs. A schematic overview of the principal hybrid‐MDV engineering approaches and the associated biological benefits is provided in Figure 5.

**FIGURE 5:**
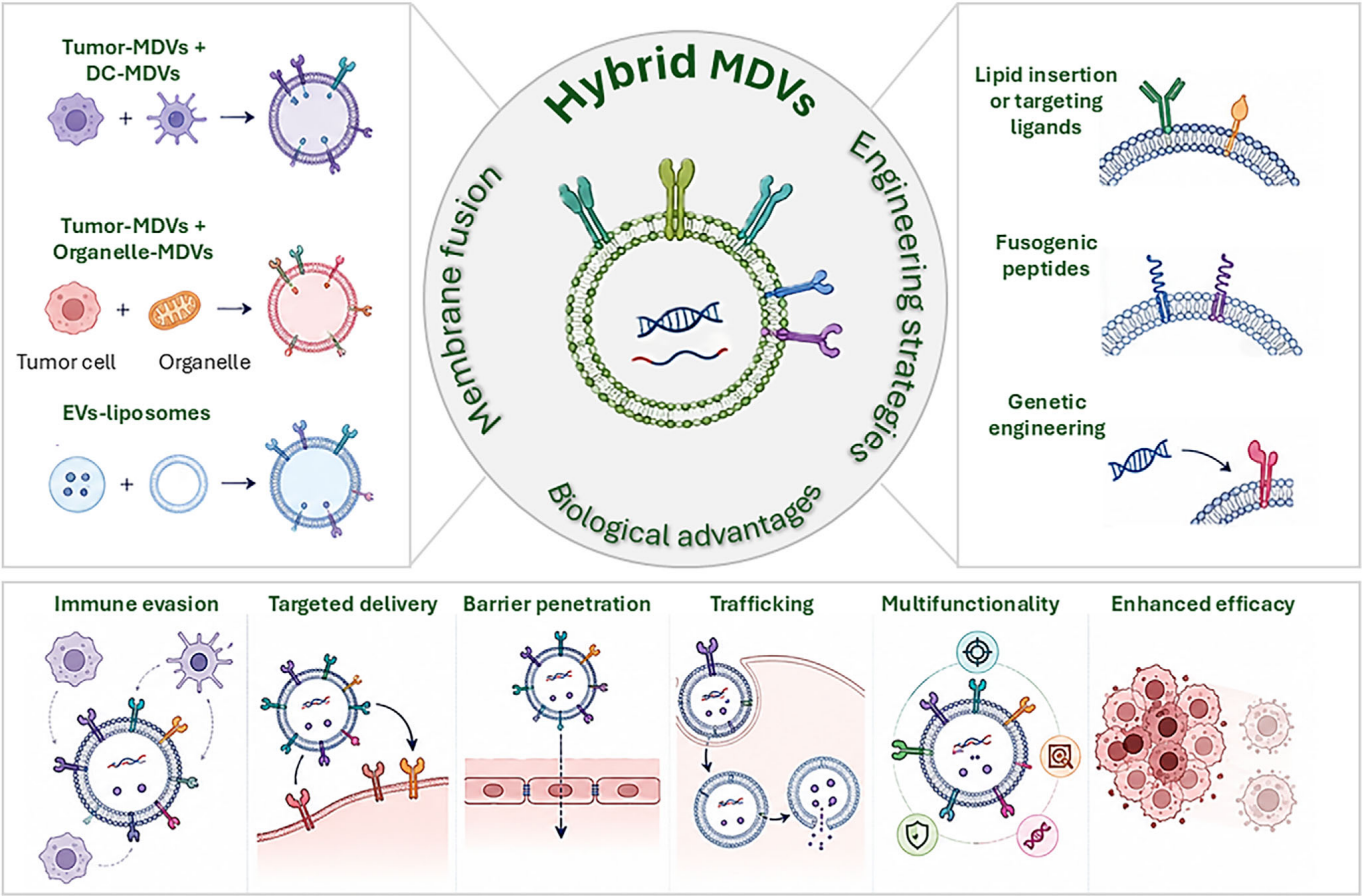
Hybrid membrane‐derived vesicles (Hybrid‐MDVs) combine biological membrane functionalities with synthetic engineering strategies to enhance therapeutic delivery. Membrane fusion, lipid insertion, fusogenic peptides, and genetic engineering enable multifunctionality, targeted delivery, and improved intracellular trafficking. These biomimetic nanoplatforms exhibit prolonged circulation through immune evasion, enhanced tissue and tumor targeting, efficient biological barrier penetration, improved endosomal escape, and therapeutic capabilities, ultimately resulting in superior efficacy and treatment outcomes.

To enhance performance and obtain multifunctionality, hybrid‐MDVs can be engineered by fusing cell membranes derived from different sources, thereby preserving ligand affinities of the parent cells [92, 93]. For example, Wen et al. wrapped porphyrin‐based ZrMOF (PCN224) by fusing cell membranes derived from both tumor cells and DCs to treat tumors [177]. The obtained hybrid MDVs, retaining homologous antigens of tumor cells and the specific tumor recognition antigens of DCs, showed significant anti‐tumor effects.

The advantages of membrane hybridization become even more evident when complex biological barriers need to be overcome. Glioblastoma‐derived MDVs already display an intrinsic ability to cross the BBB, and this property can be further expanded by incorporating organelle‐derived membranes.

In this scenario, Zou et al. proposed cancer cell‐mitochondria hybrid membrane‐camouflaged NPs encapsulating Gboxin, an inhibitor of oxidative phosphorylation, to induce apoptosis in the tumor [178]. Following BBB penetration, these hybrid vesicles selectively accumulated in mitochondria, where Gboxin inhibited ATP synthase, disrupted energy metabolism, and induced tumor cell apoptosis. The treatment significantly suppressed tumor growth and prolonged survival in orthotopic GBM models [178].

Along the same line, semisynthetic EV‐liposome hybrid‐MDVs combine the loading efficiency and formulation flexibility of liposomes with prolonged circulation and intrinsic targeting abilities of EVs [85], providing an effective platform for specific siRNA delivery [85].

Beyond membrane hybridization, these systems can be further tailored through membrane engineering. Physical approaches, such as lipid insertion, allow the incorporation of targeting ligands or fusogenic molecules without compromising membrane protein activity [179, 180].

In addition, short amino acid sequences able to promote membrane fusion have been employed to enhance intracellular delivery. These peptides, derived from naturally occurring viral proteins, such as the HA2 subunit of influenza hemagglutinin, or synthetic analogues like GALA (Glutamic acid‐Alanine‐Leucine‐ Alanine), KALA (Lysine‐Alanine‐Leucine‐Alanine), and RALA (Arginine‐Alanine‐Leucine‐Alanine), facilitate endosomal escape and cytosolic release of cargo [181]. As an example, Soprano et al. developed fusogenic cell‐derived NVs (FCSMs) by intercalating aromatic labelled and cationic lipids, like DOPE‐647N and DOTAP, within tumor cell membranes [182]. The positive charge introduced by DOTAP allowed an increase in the electrostatic interaction between the NVs and the negatively charged glycocalyx of the cell membrane, while the presence of a neutral lipid with a cone‐like structure, such as DOPE, allowed membrane destabilization [183]. Consequently, these FCSMs efficiently delivered CRISPR/Cas9 ribonucleoproteins into target cells, combining natural homotypic targeting with improved transfection performance [182].

Cell‐MDVs can be functionalized via lipid insertion to display targeting ligands such as anti‐PD‐L1 antibodies without impairing their function. In PLT‐based systems, a PDL1‐modified membranes enhance post‐surgical anti‐tumor immunity by blocking the PD‐1/PD‐L1 axis and promoting local immune activation. PLTs are particularly effective carriers due to their natural affinity for tumor sites and injured vasculature. Lipid insertion therefore represents a simple and efficient strategy to generate hybrid biomimetic platforms for targeted immunomodulation [82, 87, 131, 184, 185].

Besides improving targeting, membrane engineering can also prolong systemic circulation by reducing opsonization and phagocytic clearance. Traditionally, polyethylene glycol (PEG) is commonly used to sterically reduce the interaction of proteins with the NP surface, increasing circulation time by preventing macrophage uptake. Indeed, the PEGylation of proteins and oligonucleotides has been widely used, and it has been approved by the FDA [186, 187]. However, concerns regarding PEG‐associated immunogenicity have stimulated the development of alternative shielding polymers, including poly(N‐vinylpyrrolidone), poly(oxazoline), poly(N‐propionylmethacrylamide), poly(vinyl alcohol), polyglycerol, and polyzwitterions [187]. A recently proposed alternative is PASylation, which replaces synthetic polymers with intrinsically disordered polypeptide chains composed of repeating proline, alanine, and serine (PAS) residues, providing PEG‐like biophysical properties while potentially reducing immunogenicity [188, 189].

Furthermore, chemical modification of MDVs is commonly achieved via covalent bonds, which ensure enhanced stability for functional attachments [190]. Different molecules exposed on the cell membrane provide several potential sites for modification, such as carboxylic acids, thiols, and primary amines, which allow covalent bonds for the desired adducts [191].

Primary amines and thiol groups are the main reactive sites exploited for covalent membrane functionalization [191]. Primary amines readily form stable amide bonds with activated carboxylic acids and have been used by Patel et al. to introduce maleimide (Mal) groups onto bacterial MDVs through Mal‐PEG4‐NHS conjugation [192]. These maleimide groups can subsequently react with thiol‐containing molecules, enabling site‐specific functionalization. Accordingly, Cheng et al. employed the bifunctional linker NHS‐PEG2‐maleimide to first react with membrane amines and then anchor a thiol‐terminated E‐selectin‐binding peptide onto cell‐MDVs, improving receptor targeting while maintaining controlled binding affinity and kinetics [193]. Alternatively, membrane functionalization can also be achieved through high‐affinity noncovalent interactions, such as the biotin‐avidin system, which has been successfully applied to RBC–MDVs to enhance active targeting [194].

In contrast to physical and chemical modification strategies, which rely on post‐isolation manipulations of membranes, metabolic engineering introduces functional features during membrane biosynthesis. This strategy mainly relies on glycoengineering, which exploits the fucose salvage pathway using monosaccharides such as N‐acetylmannosamine (ManNAc), GalNAc, and fucose, and lipid engineering, which employs choline analogues to expose biorthogonal handles, including azide (N_3_) and bicyclo[6.1.0]nonane (BCN), for selective bioorthogonal targeting [195, 196, 197]. Exploiting the natural glycophysiological pathway, Harris et al. developed N3‐labelled T cell membrane‐encapsulated ICG‐PLGA NPs (N3‐TINPs), which can specifically target natural antigens and BCN artificial receptors on tumors through immune recognition and bioorthogonal chemistry [197]. Compared with unlabelled TINPs, N_3_‐TINPs achieved a 1.5‐fold increase in tumor fluorescence, improved photothermal efficacy, and reduced off‐target effects [197]. Metabolic engineering includes gene editing, which enables the cell membrane to express the desired product via transcription and translation. Recent advances in CRISPR/Cas9 technology have made this process more efficient and accurate [198]. As a result, cell‐MDVs functionalized by genetic modification show a significant increase in circulation time compared to wild‐type ones.

Genetic engineering can also promote the membrane expression of high‐affinity ligands, enhancing targeting capabilities. For instance, the VLA‐4/VCAM‐1 interaction, upregulated during inflammation, has been exploited for targeted nanoparticle delivery [199]. Engineered cells expressing the entire VLA‐4 heterodimer (α4β1) have been used to derive membranes for coating dexamethasone‐loaded nanoparticles, resulting in enhanced accumulation in inflamed endothelial tissues via this specific binding mechanism [199]. Overall, gene modification offers a promising strategy to develop “universal” biomimetic nanoparticle membranes, particularly when combined with the knockdown of immunogenic markers such as MHC I and II [200]. However, its clinical translation remains limited by technical complexity and challenges in achieving stable gene expression.

In parallel, inorganic nanomaterials such as gold nanoparticles, quantum dots, and magnetic systems have also been combined with nuclear targeting peptides (e.g., TAT, SV40 NLS) for efficient intracellular and nuclear delivery [201, 202]. Nuclear translocation occurs through importin‐mediated transport through the NPC and is known to be strongly size‐dependent, with optimal transport generally found for nanoparticles in the size range of ∼25–50 nm, while larger systems tend to display reduced efficiency. This size limitation, together with surface functionalization, is an important determinant for intranuclear accumulation, ultimately improving controlled cargo release and anticancer efficacy [203].

Overall, these observations indicate that hybrid‐MDVs outperform non‐functionalized MDVs not through a single dominant mechanism, but by integrating multiple biologically evolved solutions for immune evasion, targeting, uptake, intracellular trafficking, and endosomal escape. Table 3 summarizes the main surface modification strategies for biomimetic NVs, highlighting their technical approaches, functional advantages, and applications in gene delivery. Despite their extraordinary functional versatility, hybrid and functionalized MDVs introduce additional layers of complexity in manufacturing, characterization, and regulatory assessment, which currently represent the main barriers to large‐scale clinical translation.

**TABLE 3:** Engineering strategies applied to hybrid‐MDVs and their corresponding functional advantages in gene delivery systems.

Type of modification	Description/technical approach	Main functional advantage	Application in gene delivery	References
Dual membrane coating	Fusion of two cellular membranes (*e.g*., canc[178,204]er + dendritic cell) on a lipidic or polymeric core	Combines tumor‐homing with immune stimulation	DNA/mRNA vaccines	[178, 204]
Lipid insertion	Incorporation of fusogenic or pH‐sensitive lipids (DOPE, DOTAP, DLinMC3‐DMA) into the membrane	Enhances endosomal escape and membrane fusion	siRNA/CRISPR systems	[178, 180, 182]
Ligand anchoring	Surface insertion of peptides, antibodies, or aptamers recognizing specific receptors	Enables active targeting of tissues or cell types	miRNA/plasmid DNA delivery	[145, 202]
PEGylation and alternative stealth polymers	Surface modification with PEG or alternatives such as PAS, PVP, or zwitterionic polymers	Improves colloidal stability, prolongs blood circulation, reduces opsonization, and RES clearance	Systemic mRNA or siRNA delivery	[193, 205]
Surface charge modulation	Adjustment of zeta potential through lipid or polymer composition tuning	Improves cellular uptake and controls electrostatic interactions	Plasmid DNA/CRISPR delivery	[182, 183]
Endogenous protein decoration	Reincorporation or overexpression of natural membrane proteins (e.g., CD47)	Promotes immune evasion and self‐recognition	mRNA/siRNA delivery	[169]
Metabolic engineering	Genetic or metabolic manipulation of the parent cells to overexpress desired lipids, receptors, or proteins	Customizes membrane composition in vivo and enhances functionalization efficiency	Personalized gene therapy/cell‐derived membrane factories	[198, 199]
Magnetic or metallic nanocomposite integration	Embedding magnetic or gold NPs into the hybrid core or bilayer	Enables imaging traceability, magnetic targeting, or photothermal activation	DNA/siRNA theranostic delivery	[146, 201]

## 8 Engineering Immune Cells Through Biomimetic Approaches

Several strategies have been explored to engineer immune cells since these kinds of cells present themselves with intrinsic therapeutic and defence properties. One key obstacle is to transfect immune cells while maintaining the balance between immune activation and immunosuppression that is fundamental to preserving their functionalities in TME. To this aim, biomimetic approaches offer a powerful strategy to overcome these issues [206].

Till now, there are few studies concerning genetic delivery through MDVs, in contrast to drug delivery. YU et al. exploited the natural affinity of neutrophils for inflammatory microenvironments to deliver drugs to cancer sites. They engineered activated neutrophils through NPs conjugated with anti‐CD11b antibodies, allowing for targeted drug delivery to the tumor site [207]. Similarly, Guo et al. developed a biomimetic nanovaccine formulation to efficiently target antigens to antigen‐presenting cells (APCs), especially DCs, to induce an antigen‐specific T cell response. RBC membranes were modified with mannose (RBCM) to enhance active targeting toward APCs within the lymphatic system, exploiting mannose receptor‐mediated uptake to improve cellular recognition and uptake efficiency [208]. Additionally, DCs naturally interact with T cells, triggering their activation. Thus, using mature DC membranes to coat NVs offers a promising approach for directly targeting T cells through peptide/MHC complexes, costimulatory molecules, integrins, and adhesion molecules [209]. In this context, Li et al. developed a biomimetic vaccine based on cancer cell membranes coated on silicon@Au cores (CCM@(PSiNPs@Au)) which promoted efficient uptake of tumor antigens by DCs and induced their maturation, thereby eliciting robust antitumor immune responses. When combined with immune checkpoint blockade (ICB) therapy, this system further enhanced tumor growth inhibition and suppressed metastasis in solid tumor models [210]. In addition to T cell activation, a reprogramming of macrophages has also emerged as an important immunotherapeutic approach. Wei et al. reported a platform for re‐polarization of tumor‐associated macrophages (TAMs) from an immunosuppressive M2‐like phenotype to a pro‐inflammatory M1‐like phenotype. In their system, PLGA NPs loaded with resiquimod or DOX were electrostatically complexed with non‐pathogenic *E. coli* using glycol chitosan, which resulted in preferential accumulation in hypoxic regions of the tumor. The released cargo after macrophage phagocytosis efficiently drove M2‐to‐M1 repolarisation, contributing to tumor suppression [211]. Recently, a similar approach was adopted for the delivery of genetic cargo. TAMs were reprogrammed into an anti‐tumor M1‐like phenotype by developing functionalized NVs derived from *E. coli* protoplasts to effectively encapsulate the gene editing tool Cas9. NVs were functionalized with pH‐responsive PEG‐conjugated phospholipid derivatives (DHP) and galactosamine ligands (DGA). This resulted in efficient TAM repolarization toward an M1‐like phenotype and achieved approximately 80% tumor growth inhibition in mouse models [171]. In addition to bacterial membranes, recent studies employed cell‐MDVs to deliver mRNAs. Researchers investigated whether DCs’ membrane‐coated polyethylenimine/mRNA NPs could effectively deliver mRNA to DCs and induce immune responses. Results showed that these NPs had a higher uptake than both RBC‐coated and non‐coated NPs [212]. Another safe and effective strategy to deliver genetic materials to immune cells is phospholipid‐derived NPs that act as immunostimulatory agents. In particular, a research group combined phospholipid/glycolipid NPs with OX40‐mRNA, in order to increase the expression of this costimulatory domain in tumor‐infiltrating T cells and to stimulate antitumor immunity [213]. Further strategies investigate the combination of natural delivery vehicles, such as EVs, with cationic lipids. For instance, Klyachko et al. achieved efficient transfection through EVs loaded with plasmid DNA, mRNA, or siRNA. Since EVs’ surface is negative, they modify the membrane with multivalent cationic lipids through hydrophobic interactions to bind negatively charged genetic materials [214]. Despite the uphill road in immune cell engineering, these studies show promising tools to bypass classical transfection methods.

## 9 Challenges and Clinical Translational Perspectives of Biomimetic NVs for Gene Delivery

Besides the benefits provided by the employment of biomimetic NVs in gene delivery, critical aspects must be considered for clinical translation. The first challenge is the manufacturing process. Actually, there is no standardized and universally accepted protocol to isolate, purify, and preserve EVs, MDVs, and BMVs. The purity of the membrane could be influenced by the extraction process itself, as mechanical or chemical treatments may compromise their integrity, leading to the co‐isolation of intracellular contaminants [196]. Incomplete removal of cytosolic proteins, nucleic acids, organelle fragments, or damage‐associated molecular patterns (DAMPs) could result in immunogenic debris that can activate complement and off‐target inflammatory responses in vivo [215].

Furthermore, maintaining the correct orientation of the cell membrane is essential to ensure its biological function. In addition to common challenges shared across all cell types, such as long‐term stability, batch‐to‐batch reproducibility, and production costs, protocols must also be tailored according to the specific characteristics of the source cells [89]. RBC‐MDVs provide advantages such as long circulation time and immune evasion. However, extracting their membranes without causing protein denaturation is particularly challenging. Moreover, RBC membranes have limited protein diversity, resulting in the lack of specific targeting capabilities [216]. PLT‐MDVs face other difficulties, as PLTs are more fragile and prone to premature clotting during manipulation due to activation [217]. Likewise, membranes derived from leukocytes and cancer cells require preservation of their complex receptor composition while minimizing the risk of unwanted immune activation [218, 219].

EVs’ ability to carry genetic and protein cargo and to interact with target cells makes them highly promising therapeutic vehicles, although obtaining large amounts of high‐purity EVs remains difficult, given the inefficiency and time‐consuming isolation techniques [99, 220]. Methods like size‐exclusion chromatography can enhance the purity of EVs, but stability remains a concern, as they may lose functional activity during storage, therefore compromising therapeutic efficacy [221]. Surface engineering of EVs is a strategy to enhance targeting specificity, but certain cell‐derived EVs may still induce undesired immune responses, raising safety concerns [222].

The employment of bacterial membranes also brings both potential and complexity. Their strong immunostimulatory properties make them powerful candidates for vaccine development, yet these same properties can lead to excessive immunogenicity, mainly due to the presence of LPS [223]. Furthermore, isolating an intact bacterial membrane without contamination from intracellular components is a technically challenging procedure [166].

A major bottleneck in MDV‐based delivery is the loading of nucleic acids, since most conventional encapsulation approaches can compromise vesicle integrity or alter their native physicochemical properties [224, 225]. Most common loading techniques such as electroporation, extrusion, sonication, and freeze‐thaw processing may improve cargo entry, but they often induce membrane perturbation, lipid damage, protein unfolding, or cargo aggregation, thereby affecting vesicle stability and biological performance [226]. Chemical loading strategies, despite avoiding mechanical stress, introduce additional concerns related to residual reagents and potential toxicity [227]. Moreover, nucleic acid encapsulation efficiency is strongly influenced by formulation parameters and loading conditions, which often leads to poor reproducibility and cargo loss during storage [228, 229, 230].

Additional biological barriers further limit therapeutic efficacy. Membrane coatings alone do not significantly improve endosomal escape unless combined with complementary functional strategies [231]. Moreover, the in vivo biodistribution and clearance of MDVs remain unpredictable, with potential off‐target accumulation and rapid clearance by macrophages [232].

Immune compatibility also deserves careful consideration. Although membrane coatings are designed to mimic “self”, allogeneic membranes or improperly processed proteins may still be recognized by the immune system. Structural alterations introduced during membrane preparation can generate neoantigens, activate complement, and accelerate macrophage‐mediated clearance [233, 234]. Additionally, repeated dosing may trigger immune memory against the membrane, leading to faster clearance and reduced therapeutic benefit [235]. These concerns highlight the importance of carefully controlling membrane purity, choosing the source material wisely, and ensuring consistent formulation reproducibility.

Key challenges, such as scalability, variability of donor source cells, and conventional extraction methods based on ultracentrifugation, cause batch‐to‐batch variability [236, 237]. In recent years, innovations such as microfluidic extrusion, tangential flow filtration, and three‐dimensional bioreactors have improved purity and yield, becoming closer to good manufacturing practice (GMP) standards [233, 238]. More than 100 clinical trials are evaluating natural or engineered EVs for inflammatory, oncological, and pulmonary diseases, with products like ExoFlo, AGLE‐102, and RION PEP already in phase I/II studies [239]. Compared with EVs, MDVs are typically more uniform in size, morphology, and surface chemistry, which supports better reproducibility at large scale [240]. Furthermore, their integration with synthetic lipids and polymers has enabled hybrid biomimetic platforms with improved stability, circulation time, and targeting performance, offering a promising strategy to bridge the advantages of biological and synthetic delivery systems [241, 242].

## 10 Conclusions

In this review, we propose MDVs and hybrid‐MDVs as a new generation of biomimetic non‐viral gene delivery systems, merging the biological complexity of natural membranes with the versatility of synthetic nanotechnology. These platforms can overcome many limitations of conventional delivery strategies by exploiting cell‐specific functions while maintaining the tunability of engineered nanocarriers. Beyond technological optimization, the field has future questions to answer. Further insights into the connection between membrane composition and biological performance are required to establish rational design principles for biomimetic carriers. Likewise, defining the effect of different membrane sources on biodistribution, intracellular trafficking, and therapeutic outcomes across disease settings will be critical to inform the choice of the most suitable platform for each application.

## Author Contributions

**Clara Baldari**: conceptualization, writing – original draft, writing – review and editing. **Claudia Leone**: writing – original draft, writing – review and editing. **Gabriella Leccese**: writing – original draft, writing – review and editing. **Claudia De Stradis**: writing – original draft, writing – review and editing. **Alessandra Invidia**: writing – original draft, writing – review and editing. **Concetta Quintarelli**: writing – review and editing. **Biagio De Angelis**: writing – review and editing. **Franco Locatelli**: writing – review and editing. **Giuseppe Gigli**: writing – review and editing, funding acquisition. **Gabriele Maiorano**: writing – review and editing, funding acquisition. **Ilaria Elena Palamà**: conceptualization, supervision, writing – review and editing.

## Funding

This work was funded by “Tecnopolo per la medicina di precisione” (TecnoMed Puglia)—Regione Puglia: DGR n. 2117 del 21/11/2018, EU funding within the MUR PNRR “National Center for Gene Therapy and Drugs based on RNA Technology” (Project no. CN00000041 CN3 RNA), PNC Italian Health Ministry—Hub Life Science—Terapia Avanzata (LSH‐TA) PNC‐E3‐2022‐23683269, PNC Italian Health Ministry PNRR‐MR1‐2022‐12376725, “Preclinical Development of Gene Therapy for Dystrophic Epidermolysis Bullosa”, EU European Union—Next Generation PRIN PNRR 2022‐ P2022EPK9B “Nanodelivery of Slpi Prevents Inflammatory‐associated UC Development in the Spontaneous Model Winnie”, and Project “Biotecnologia, Bioinformatica e Sviluppo farmaceutico” per la creazione di un hub delle scienze della vita, piano operativo salute (Piano di Sviluppo e Coesione del Ministero della Salute 2014‐2020), Traiettoria 4, Azione 4.1‐ Cod. T4‐AN‐01.

## Conflicts of Interest

The authors declare no conflicts of interest.

## Data Availability

The authors have nothing to report.
